# Determination of lesinurad in rat plasma by a UHPLC–MS/MS assay

**DOI:** 10.1186/s13065-017-0353-6

**Published:** 2017-11-28

**Authors:** Xiao-Yang Zhou, Ling-Jing Yuan, Zhe Chen, Peng-Fei Tang, Xiang-Yu Li, Guo-Xin Hu, Jian-Ping Cai

**Affiliations:** 1 The MOH Key Laboratory of Geriatrics, Beijing Hospital, National Center of Gerontology, Beijing, 100730 People’s Republic of China; 2 Department of Pharmacology, School of Pharmacy, Wenzhou Medical University, Wenzhou, 325035 Zhejiang China

**Keywords:** Lesinurad, UHPLC–MS/MS, Rat plasma, Pharmacokinetics

## Abstract

Lesinurad is an oral inhibitor of urate-anion exchanger transporter 1 and has been approved by the US Food and Drug Administration for combination therapy with a xanthine oxidase inhibitor for the treatment of hyperuricemia associated with refractory gout. In the present study, a sensitive and specific ultra high-performance liquid chromatography with tandem mass spectrometry assay was established and verified for the determination of lesinurad in rat plasma and was described in details for the first time. Chromatographic separation of lesinurad and diazepam (internal standard, IS) was performed on a Rapid Resolution HT C18 column (3.0 × 100 mm, 1.8 µm) using methanol–water (70:30, v/v) as the mobile phase at a flow rate of 0.3 mL/min. Lesinurad and IS were extracted from plasma by liquid–liquid extraction using ethyl acetate. The mass spectrometric detection was carried out using an electrospray ionization source in positive mode. Multiple reaction monitoring was used for quantification of the precursor to product ion at *m/z* 405.6 → 220.9 for lesinurad and *m/z* 285.1 → 192.8 for IS. The assay was well validated for selectivity, accuracy, precision, recovery, linearity, matrix effects, and stability. The verified method was applied to obtain the pharmacokinetic parameters and concentration–time profiles for lesinurad after oral/intravenous administration in rats. The study might provide an important reference and a necessary complement for the qualitative and quantitative evaluation of lesinurad.

**Electronic supplementary material:**

The online version of this article (10.1186/s13065-017-0353-6) contains supplementary material, which is available to authorized users.

## Introduction

Gout is a metabolic disorder resulting from the deposition of urate crystals caused by altered purine metabolism leading to hyperuricemia. It has various clinical manifestations, including arthritis, soft-tissue masses (i.e., tophi), nephrolithiasis, and urate nephropathy, which occur because of the deposition of monosodium urate crystals in the joints, soft tissues, and kidneys. Gout prevalence in the USA was 3.9% in 2007–2008 [1], 2.49% in the UK in 2012 [2], and 1.1% in mainland China [3]. Epidemiological studies suggest that there has been a rise in the prevalence of gout over recent decades. Gout and hyperuricemia are associated with hypertension, metabolic syndrome, and cardiovascular diseases [4–7].

Uric acid is the final oxidation product of purine metabolism. Urate homeostasis depends on the balance between production, intestinal secretion, and renal excretion [8]. It has been estimated that approximately one-third of total urate disposal is by intestinal uricolysis and two-thirds are by urinary uric acid excretion involving secretion and reabsorption in the kidney tubules [7, 9, 10]. Hyperuricemia may be caused by either overproduction or underexcretion of uric acid. It is generally accepted that decreased efficiency of renal uric acid excretion is primarily responsible for hyperuricemia in the majority of gout patients [7].

**Fig. 1:**
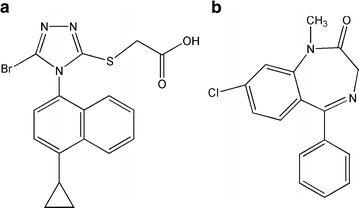
Chemical structures of **a** lesinurad and **b** diazepam (IS)

Lesinurad (Fig. 1), a newer drug to treat hyperuricemia associated with refractory gout that functions by targeting the urate-anion exchanger transporter (URAT1), was approved by the US Food and Drug Administration (USFDA) in December 2015 [11, 12], for combination therapy with a xanthine oxidase inhibitor. It was also approved by the European Medicines Agency’s Committee for Medicinal Products for Human Use for this clinical indication throughout the European Union in February 2016 [13]. URAT1, a transmembrane protein that serves as a highly urate-specific and organic anion exchanger, is localized to the luminal membrane of the proximal tubular epithelial cells [14]. All or nearly all uric acid is freely filtered at the glomerulus and most of the filtered urate is reabsorbed in the proximal tubule through URAT1. Lesinurad functions as a selective uric acid reabsorption inhibitor by inhibiting URAT1 and organic anion transporter 4 (OAT4), and so increases the urinary excretion of uric acid [15, 16].

The previously studies primarily focused on descriptions of pharmacokinetics and pharmacodynamics of lesinurad in healthy individuals or gout patients under given different therapeutic regimes. In these researches, the determinations of lesinurad in plasma were all performed by Ardea Biosciences (San Diego, CA, USA) using high-performance liquid chromatography–tandem mass spectrometry/mass spectrometry (HPLC–MS/MS) and their methods were not elaborated at all [17–20]. The aim of this study was to develop and elaborate on a sensitive and validated UHPLC–MS/MS method for the quantitative evaluation of lesinurad in rat plasma samples. The validation of this method was also performed, taking into account the selectivity, sensitivity, accuracy, precision, linearity, recovery, and stability, and the method was then implemented to estimate and determine the pharmacokinetic properties of lesinurad. Our data was intend to provide an important reference and a necessary complement for the assay for the determination of lesinurad.

## Methods

### Reagents and materials

Lesinurad was purchased from Toronto Research Chemicals (Toronto, Canada) and diazepam (internal standard, IS) was obtained from Sigma (St. Louis, MO, USA). HPLC-grade methanol, formic acid, and ethyl acetate were purchased from Merck Company (Darmstadt, Germany). The water used throughout the study was obtained from a Milli-Q Reagent Water System (Millipore, Billerica, MA, USA).

### UHPLC–MS/MS analysis

Plasma samples were analyzed by the LC–MS/MS method. The system was composed of an Agilent 1290 LC system (Agilent Technologies, Santa Clara, CA, USA) with a 1.8 μm Rapid Resolution HT C18 column (3.0 × 100 mm, Agilent Technologies) coupled to an Agilent 6490 Triple Quadrupole mass spectrometer (Agilent Technologies) equipped with an electrospray ionization (ESI) source. The mobile phase consisted of methanol–water (70:30, v/v). The flow rate was 0.3 mL/min and the injection volume was 5 µL. The total run time was 5 min. Under the above conditions, lesinurad and diazepam (IS) were well separated and their retention times were 2.90 and 3.57 min, respectively. For the determination of lesinurad and IS, the positive-ion mode was used according to the conditions shown in Table 1. A dynamic multiple reaction monitoring (MRM) method was performed to identify the specific precursor and product ions of the lesinurad and IS inside their retention time windows. The capillary voltage was set to 4.0 kV in positive mode and the nebulizer pressure was set to 15 psi. The gas temperature was set to 300 °C at a flow rate of 6 L/min.

**Table 1:** MS parameters for lesinurad and diazepam

Compound name	Precursor ion (*m/z*)	Product ion (*m/z*)	Collision energy (eV)	Fragmentor voltage (V)
Lesinurad	405.6	220.9	35	380
Diazepam	285.1	192.8	32	380

### Sample preparation

HCl (1 M, 50 µL) and ethyl acetate (1000 µL) were added to samples of rat plasma (100 µL) and diazepam (1 µg/mL, 20 µL) was added as an internal standard. The tube was thoroughly mixed by vortexing for 2 min. After centrifugation at 13,000*g* for 10 min, the organic phase was transferred to a new clear tube and evaporated to dryness under a nitrogen stream at 45 °C. The dried samples were dissolved in the mobile phase (100 µL) and used for the LC–MS/MS analysis.

### Calibration standards and quality control samples

The stock solutions of lesinurad were dissolved in dimethyl sulfoxide (DMSO) to make the calibration standards. Working solutions of lesinurad for calibration and controls were prepared from the corresponding stock solutions by dilution with methanol. The lesinurad calibration standards were prepared by adding 5 µL of the working solution to 95 µL of the blank rat plasma. The calibration plots were carried out with various final concentrations (50, 100, 250, 1000, 5000, 10,000, 50,000 ng/mL) of lesinurad calibration standards with appropriate amounts of the working standard solution of IS in rat plasma. The stock solution of IS was dissolved in methanol to a final concentration of 1 µg/mL. Quality control (QC) samples were prepared by the same method as the calibration standards at three different concentrations (100, 1000, and 25,000 ng/mL). All of the solutions were stored at − 20 °C and brought to room temperature before use.

### Method validation

Method validation was carried out according to the United States Food and Drug Administration (USFDA) guidance for bioanalytical method validation [21]. Validation was performed for specificity, linearity, accuracy and precision, matrix effects and stability.

#### Selectivity and specificity

Selectivity is the ability of an analytical method to differentiate and quantify the analyte in the presence of other sample components [21]. The method selectivity was verified by analyzing blank plasma samples from six rats, blank samples spiked with lesinurad and IS, and rat plasma samples. The degree of interference was assessed through comparison of the chromatograms of blank plasma with the chromatograms of plasma spiked with lesinurad and IS.

#### Accuracy, precision and recovery

QC samples at three concentrations (100, 1000, 25,000 ng/mL) and LLOQ samples (50 ng/mL) in rat plasma (*n* = 6) were analyzed repeatedly over three separate days. Relative standard deviation (RSD %) and relative error (RE %) were calculated to assess the accuracy and precision of the method. Recovery experiments revealed the extraction efficiency of the analytical method and were performed by comparing the peak areas of extracted QC samples at three concentrations with those of unextracted standards at the same concentrations in post-extracted blank plasma (*n* = 6).

#### Linearity and lower limit of quantification

Calibration curves were constructed by measuring calibration samples at seven different concentrations (50–50,000 ng/mL) on three separate days. The lowest concentration of lesinurad in the calibration curves that could be reproducibly quantified with precision (< 20%) and accuracy (80–120%) was accepted as the lower limit of quantification (LLOQ). Additionally, the analyte signal of the LLOQ sample should be at least five times the signal of a blank sample.

#### Matrix effects

Six different blank rat plasma samples were extracted and spiked with the QC samples at three concentrations (10, 1000, and 25,000 ng/mL). The ratios of the peak areas of the analytes added into post-extracted blank plasma and the peak areas of pure authentic standards at equivalent concentrations were measured and defined as the matrix effect (ME).

#### Stability

To evaluate the stability of the method, lesinurad levels in rat plasma were assessed using six replications at three concentrations (10, 1000, and 25,000 ng/mL). These experiments evaluated the stability of the QCs during sample collection and handling under various storage conditions and the analytical process, including freeze–thaw stability (from − 70 °C to room temperature for three cycles), short-term temperature stability (ca. 22 °C for 12 h), long-term stability (− 20 °C for 30 days), and post-preparation stability (in the autosampler at 4 °C for 48 h). RSD values of the mean test signals within 15% were regarded as indicative of stability.

### Pharmacokinetic study in rats

Twelve male Sprague–Dawley rats (330 ± 30 g) were purchased from the Laboratory Animal Center of Wenzhou Medical University (Wenzhou, China). Animal experiments were demonstrated to be ethically acceptable and were carried out according to the Guidelines of the Experimental Animal Care and Use of Laboratory Animals of Wenzhou Medical University (ethical committee approval number: wydw2016-0018). After fasting for 12 h, all rats were divided into two groups, which received lesinurad by either intragastric administration (20 mg/kg) or intravenous administration (5 mg/kg). Blood samples (ca. 0.3 mL) were collected from the tail vein into heparinized tubes at various times (0.083, 0.25, 0.5, 0.75, 1, 2, 4, 6, 8, 10, 12, and 24 h). The blood samples were centrifuged at 13,000*g* for 10 min at 4 °C and then pipetted into clean tubes and stored at − 80 °C until analysis. The pharmacokinetic parameters were calculated using DAS software (version 3.0, Shanghai University of Traditional Chinese Medicine, Shanghai, China).

## Results and discussion

### Method development

#### Chromatographic conditions

The chromatographic conditions were optimized to achieve efficient separation of lesinurad and IS with good resolution, short runtimes and symmetrical peak shapes. In this study, methanol–water (70:30, v/v) with or without 0.1% formic acid was used as the mobile phase with isocratic elution. The total chromatographic analysis run time was 5 min, with lesinurad and diazepam (IS) eluting after 2.90 and 3.57 min, respectively. The optimum peak resolution was obtained using the Rapid Resolution HT C18 column (100 × 3.0 mm diameter) with a column oven temperature of 35 °C.

#### Mass spectrometry

The mass spectrometry operating parameters, such as ESI source gas temperature, source gas flow, capillary and fragmentor voltages, ion modes, and collision energy, were optimized to obtain the optimum response and resolution of lesinurad and IS. After the optimization experiments, the following conditions were selected: gas temperature 300 °C, source gas flow 6 L/min, capillary voltage 4.0 kV in positive mode, and nebulizer pressure 15 psi (Table 1). Diazepam was selected as the IS because of its similar extraction recovery and chromatographic performance to lesinurad, and its detection sensitivity in the ESI positive-ion mode.

#### Optimization of sample extraction

The optimization of sample extraction was carried out in order to improve sensitivity and reliability of UHPLC–MS/MS assay. Protein precipitation and liquid–liquid extraction, which are common sample extraction options, were compared and optimized in the study. It was proven that ethyl acetate liquid extraction exhibited a better recovery (98.94–106.87%), and lower matrix effects as well. Consequently, ethyl acetate liquid–liquid extraction was used as plasma samples extraction method in the study. A further optimization was applied to sample treatment by evaporation of solvent under a nitrogen stream and redissolution in the mobile phase to achieve high sensitivity of the assay.

### Method validation

#### Selectivity

**Fig. 2:**
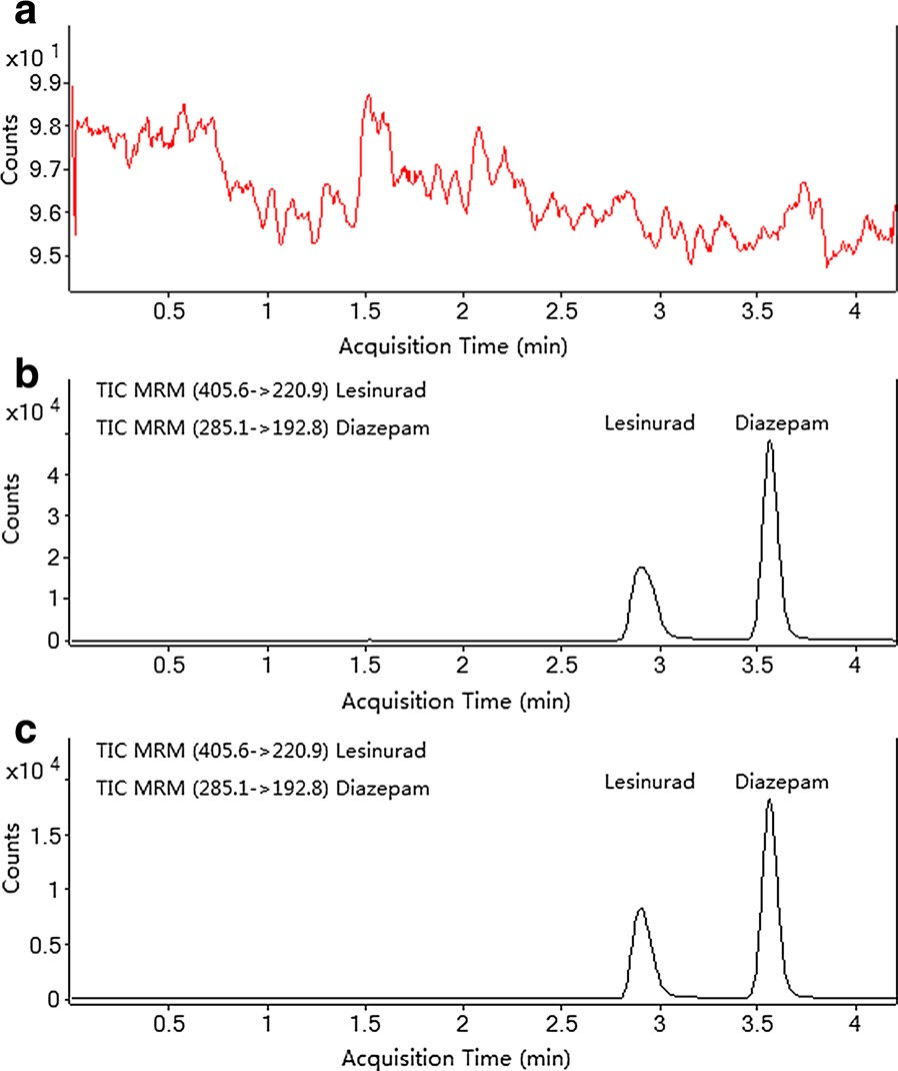
Representative UHPLC–MS/MS chromatograms of lesinurad and IS in rat plasma samples. **a** A blank plasma sample; **b** a blank plasma sample spiked with lesinurad and IS; **c** a rat plasma sample obtained 1 h after oral administration of lesinurad

Typical LC–MS/MS chromatograms of blank plasma, blank plasma spiked with lesinurad (50 ng/mL) and IS (200 ng/mL), and a rat plasma sample taken 1 h after oral administration of a single dose of 20 mg/kg lesinurad are shown in Fig. 2. There was no endogenous interference in the blank plasma at the retention time of lesinurad (2.90 min) or the IS (3.57 min).

#### Linearity and lower limit of quantification

The linearity was evaluated by linear regression of lesinurad/IS peak area ratios versus lesinurad concentrations. The assay was identified to be linear with a correlation coefficient (R^2^) of 0.998 in the range of 50–50,000 ng/mL for lesinurad in rat plasma. The lowest concentration on the standard curve was recognized as the LLOQ (50 ng/µL) for this assay. The bioavailability of lesinurad was 57.36%. Compared with previous study, the LLQQ identified in our study was lower than that applied for determination of lesinurad in human plasma [18]. Our further experiments were carried out and showed that the limit of quantitation (LOQ) of this assay was 0.5 ng/ml (Additional file 1: Figure S1).

#### Precision and accuracy

QC samples at three concentration levels (100, 1000, and 25,000 ng/mL) and LLOQ samples were analyzed to determine the accuracy and precision of the method. The results are shown in Table 2. The intra-day and inter-day precision values (RSD %) were ≤ 8.25 and ≤ 7.79%, respectively. The intra-day and inter-day accuracy values were in the ranges of 93.98–101.93 and 93.23–102.93%, respectively, compared to the true values. The analysis proved that the present method exhibits good accuracy and precision.

**Table 2:** Precision, accuracy, recovery, and ME for lesinurad for samples in rat plasma (*n* = 6)

Concentration (ng/mL)	Intra-day				Inter-day				Recovery (%)	ME (%)
	Mean ± SD	RSD (%)	RE (%)	Accuracy (%)	Mean ± SD	RSD (%)	RE (%)	Accuracy (%)		
50	54.81 ± 4.6	8.46	4.88	109.62	56.70 ± 3.6	6.38	2.85	113.4	–	–
100	93.98 ± 7.8	8.25	3.37	93.98	99.26 ± 6.4	6.43	2.64	99.26	106.87	101.95
1000	942.97 ± 64.4	6.83	2.79	94.30	932.26 ± 11.6	1.24	0.05	93.23	98.94	109.19
25,000	25,481.66 ± 525.4	2.06	0.84	101.93	25732.98 ± 2005.4	7.79	0.01	102.93	101.12	107.21

#### Recovery and matrix effects

The recovery and MEs of lesinurad at three different concentrations (100, 1000, and 25,000 ng/mL) are presented in Table 2. The recoveries of lesinurad were 98.94–106.87% and the MEs were in the range of 101.95–109.19% (< 15%). The recovery and MEs for IS (200 ng/mL) were 108.76 and 99.42%, respectively, compared to the true values. The results indicated that the recovery of lesinurad by liquid–liquid extraction was feasible and consistent, and that the plasma had little effect on the response of the lesinurad signal.

#### Stability

The stability data for lesinurad at three different concentrations (100, 1000, and 25,000 ng/mL) in rat plasma under various conditions are shown in Table 3. The REs were < 15% of their true values. These results demonstrated that lesinurad was stable in rat plasma under a range of storage conditions (at room temperature for 12 h, at − 20 °C for 30 days, at 4 °C for 48 h, and after three freeze–thaw cycles).

**Table 3:** Stability tests of lesinurad in rat plasma under different storage conditions (*n* = 6)

Concentration (ng/mL)	Room temperature		4 °C		Freeze–thaw (3 cycles)		− 20 °C (30 days)	
	RSD %	RE %	RSD %	RE %	RSD %	RE %	RSD %	RE %
100	6.48	− 2.54	8.25	− 6.02	9.22	0.39	1.83	0.32
1000	4.82	− 6.62	6.83	− 5.70	7.82	− 10.16	6.71	− 8.77
25,000	3.41	− 4.54	2.06	1.93	8.19	6.48	7.38	8.45

### Pharmacokinetic study in rats

**Fig. 3:**
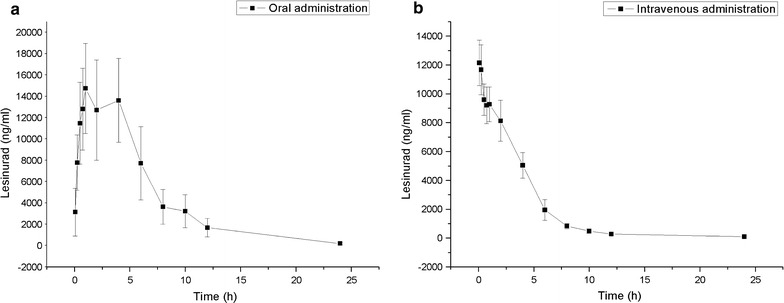
Mean plasma concentration versus time curves after oral or intravenous administration of lesinurad in rats. **a** Oral administration (20 mg/kg); **b** intravenous administration (5 mg/kg)

The validated UHPLC–MS/MS assay was applied to a single-dose pharmacokinetic study of lesinurad in male Sprague–Dawley rats. The data for the pharmacokinetic parameters of lesinurad after oral (20 mg/kg) or intravenous (5 mg/kg) administration, which were derived using non-compartmental analysis by DAS software, are summarized in Table 4. Lesinurad was found to be absorbed quickly (*T*_*max*_) and eliminated rapidly (*t*_*1/2*_). The mean plasma concentration versus time curves after oral and intravenous administration are shown in Fig. 3. A double-peak phenomenon was observed in the mean plasma concentration versus time curve after oral administration of lesinurad, which is different from the results obtained from studies in gout patients [17] or healthy adults [18, 19].

**Table 4:** The pharmacokinetic parameters of lesinurad in rat plasma after oral or intravenous administration

Parameter	Unit	Lesinurad (mean ± SD)	
		iv 5 mg/kg	po 20 mg/kg
AUC_(*0*–*t*)_	µg/L h	46,219.33 ± 5420.8	106,044.73 ± 32,137.3
AUC_(*0*–*∞*)_	µg/L h	46,541.72 ± 32,232.5	106,613.55 ± 32,232.5
t_*1/2*_	h	3.92 ± 1.6	3.22 ± 0.4
*T*_*max*_	h	0.14 ± 0.1	2.46 ± 1.7
*V*	L/kg	0.61 ± 0.2	0.94 ± 0.3
*CL*	L/h/kg	0.11 ± 0.0	0.20 ± 0.1
*C*_*max*_	µg/L	12,441.84 ± 1694.2	16,719.45 ± 2966.5
MRT_(*0*–*t*)_	h	3.39 ± 0.3	5.06 ± 0.6
MRT_(*0*–*∞*)_	h	3.58 ± 0.3	5.19 ± 0.6
Absolute bioavailability		57.36%

## Conclusions

A selective, sensitive, accurate, reliable, and reproducible UHPLC–MS/MS assay for the quantification of lesinurad in rat plasma has been established and verified. The validated assay has been successfully applied to deliver reliable data on the pharmacokinetic profile of lesinurad in rats.

## Additional file

**Additional file 1: Figure S1.** Identification for the limit of quantitation of this assay. (A) 0.25 ng/mL; (B) 0.5 ng/mL; (C) 1.0 ng/mL; (D) 2.5 ng/mL; (E) 5 ng/mL; (F) 10 ng/mL.
