# Supplementary material for: Determination of lesinurad in rat plasma by a UHPLC–MS/MS assay
Source: Chem Cent J. 2017 Nov 28;11:121. doi: 10.1186/s13065-017-0353-6 (PMC5704027; doi:10.1186/s13065-017-0353-6)
Supplement: Supplementary file 1 — Additional file 1: Figure S1. Identification for the limit of quantitation of this assay. (A) 0.25 ng/mL; (B) 0.5 ng/mL; (C) 1.0 ng/mL; (D) 2.5 ng/mL; (E) 5 ng/mL; (F) 10 ng/mL. [file 13065_2017_353_MOESM1_ESM.docx]

Additional material

**Figure S1**

Identification for the limit of quantitation of this assay. (A) 0.25 ng/mL; (B) 0.5 ng/mL; (C) 1.0 ng/mL; (D) 2.5 ng/mL; (E) 5 ng/mL; (F) 10 ng/mL.

(A)

(B)

(C)

(D)

(E)

(F)
